# Elevated Creatine Kinase and Lactic Acid Dehydrogenase and Decreased Osteocalcin and Uncarboxylated Osteocalcin are Associated with Bone Stress Injuries in Young Female Athletes

**DOI:** 10.1038/s41598-018-36982-0

**Published:** 2018-12-21

**Authors:** Takeshi Miyamoto, Yuko Oguma, Yuiko Sato, Tami Kobayashi, Eriko Ito, Mayaka Tani, Kana Miyamoto, Yuji Nishiwaki, Hiroyuki Ishida, Toshiro Otani, Hideo Matsumoto, Morio Matsumoto, Masaya Nakamura

**Affiliations:** 1 Department of Orthopedic Surgery, Keio University School of Medicine, 35 Shinano-machi, Shinjuku-ku, Tokyo 160-8582 Japan; 2 Department of Advanced Therapy for Musculoskeletal Disorders, Keio University School of Medicine, 35 Shinano-machi, Shinjuku-ku, Tokyo 160-8582 Japan; 3 Department of Department of Musculoskeletal Reconstruction and Regeneration Surgery, Keio University School of Medicine, 35 Shinano-machi, Shinjuku-ku, Tokyo 160-8582 Japan; 4 Sports Medicine Research Center, Keio University, 4-1-1 Hiyoshi, Kohoku-ku, Yokohama, Kanagawa 223-0061 Japan; 5 Faculty of Nursing and Medical Care, Keio University, 35 Shinano-machi, Shinjuku-ku, Tokyo 160-8582 Japan; 6 Institute for Integrated Sports Medicine, Keio University, 35 Shinano-machi, Shinjuku-ku, Tokyo 160-8582 Japan; 7 Department of Environmental and Occupational Health, School of Medicine, Toho University, 5-21-16 Omori-nishi, Ota-ku, Tokyo 143-8540 Japan

## Abstract

Stress fractures are a limitation for athletes not only in sports performance but in activities of daily living. Thus, preventing them is crucial. In female athletes, a triad of symptoms including low energy availability, functional hypothalamic amenorrhea and osteoporosis are considered risk factors for stress injuries, but biomarkers predictive of these outcomes are not available. Here, we evaluated 56 female university athletes and found that 13 had a history of stress bone injuries. Logistic regression analysis demonstrated that dysmenorrhea including amenorrhea, but not reduced food intake or body weight loss, was significantly associated with stress injuries. When we subdivided subjects into stress fracture and non-fracture groups, we found that serum levels of creatine kinase (CK) and lactic acid dehydrogenase (LDH) were significantly higher in the fracture group, while osteocalcin and uncarboxylated osteocalcin (ucOC), which are bone forming parameters, significantly decreased. Low vitamin D levels are associated with stress fractures, but serum vitamin D levels were higher in fracture compared to non-fracture subjects. We followed up 32 subjects for one year, and three exhibited new stress injuries during that period. A history of stress fracture history is significantly associated with experiencing a new stress fracture. We also found that subjects with new fracture performed significantly greater exercise activity than did non-fracture subjects. Taken together, our data indicate that increased serum CK and LDH and decreased serum osteocalcin and ucOC are biomarkers of stress injuries, and evaluating these markers along with dysmenorrhea, stress fracture history or high sports activity could predict future stress fractures in female athletes.

## Introduction

Sports injuries such as fractures, dislocation, and ligament and tendon ruptures are caused by high energy trauma. In contrast, stress fractures are induced by repetitive minor trauma to bone, leading to fragility. Low energy availability with or without an eating disorder, menstrual dysfunction, or low bone mineral density, which constitute a female athlete symptom triad, reportedly contribute to stress injuries^1–3^, and these factors are thought to be linked to one another. Low energy availability likely promotes functional hypothalamic amenorrhea, in turn leading to osteoporosis development, a risk for fragility fractures. Based on the Female Athlete Triad Cumulative Risk Assessment, the following are conditions of risk of stress injuries: low energy availability with or without an eating disorder, a low body mass index, delayed menarche, oligomenorrhea (6–9 periods in 12 months) or amenorrhea (<6 periods over 12 months), low BMD, and prior stress reaction or fracture. Based on the number of these parameters present, athletes can be classified as low (0–1 points), moderate (2–5 points), or high (≥6 points) risk^1,4^.

Among various sports, stress injuries often occur in running events, but they also occur in other sports-related activities^5–7^. Improvements in energy availability could be achieved by increasing energy intake or decreasing energy expenditure during exercise periods^8,9^; however, both goals are difficult for athletes to achieve due to limits on body weight. Taking low dose pills reportedly increases bone mineral density (BMD)^10^, but hormone replacement therapy is associated with an increased risk of stroke and venous thromboembolism^11^. Vitamin D also regulates bone metabolism^12^, and low vitamin D status is reportedly a risk factor for fragility fractures^13,14^. Low vitamin D levels are also associated with stress injuries^15,16^, and Vitamin D supplementation reportedly decreases occurrence of stress fracture^17^. Others report varying effects on BMD following vitamin D treatment^18^. Selective estrogen receptor modulators are used to treat post-menopausal osteoporosis patients^19,20^, but are listed as doping agents, limiting pharmacological strategies for female athletes. Moreover, serum or urinary biomarkers to predict stress injuries are currently not available.

Young female athletes often exhibit dysmenorrhea, including oligomenorrhea or amenorrhea^1,3^. Estrogen deficiency due to menopause is a risk for osteoporosis development in elderly^21^, and premature menopause also promotes osteoporosis in younger women^22^. In post-menopausal patients, osteoclastic bone resorption increases and bones acquire a high turnover state, leading to osteoporosis development. In osteoporosis patients, both bone-resorption and bone-forming parameters become elevated. Estrogen receptors are expressed in osteoclasts^23^, and loss of estrogen following menopause promotes osteoclast activation^24^. Thus, estrogen plays a key role in inhibiting osteoclastic activity and maintaining bone mass.

Here, we conducted blood and urine tests in 56 female athletes aged 18–22 years and collected information regarding their history of stress fracture and dysmenorrhea. Among subjects, 23.2% had a history of stress fracture and 46.4% reported past dysmenorrhea, outcomes significantly linked. Some (17.9%) showed vitamin D deficiency but not associated with stress injuries. Subjects in a fracture group showed higher creatine kinase (CK) and lactic acid dehydrogenase (LDH) levels and lower osteocalcin and uncarboxylated osteocalcin (ucOC) than non-fracture subjects. Thirty-two subjects were followed for a year, and three, who exercised more than others, showed new stress fractures. Previous stress facture history was significantly associated with new stress fractures. Our data suggests ways to predict stress fractures in young female athletes based on biomarker analysis combined evaluation of related symptoms.

## Materials and Methods

### Study Design and Subjects

We conducted retrospective and prospective cohort studies, both approved by the ethics committee at Keio University School of Medicine and carried out in accordance with clinical study guidelines. Subjects were 58 female student athletes at Keio University aged 18 to 22 years at first visit in 2017. Two subjects were excluded due to incomplete data. The remaining 56 played soccer (23. 2%), tennis (23.2%), or basketball (16.1%) and/or participated in running (17.9%), fencing (7.1%), yachting (3.6%), canoeing (3.6%), gymnastics (1.8%) or swimming (1.8%). Written informed consent was obtained from all. Subjects also completed a self-reported questionnaire regarding current and/or previous menstruation status, stress fracture(s), incidence of reduced food intake or weight changes during periods of rigorous exercise, smoking/alcohol habits, past history of drug usage or food intake, and daily sports-related activities.

Of the 56 subjects, 32 were followed for a year then asked to complete the same questionnaire and report current stress injuries and daily nutritional and sports activities. That data was evaluated statistically using Eiyokun software (Kenpakusya, Tokyo, Japan).

### Measurements

Body weight and height were assessed, and body mass index was calculated for all subjects. Serum and urine samples were collected at the first visit, which occurred at the end-of-term exam period in which exercise activity is generally low. The 32 subjects were then re-evaluated a year later. Complete blood cell counts including white blood cells (WBCs), red blood cells (RBCs), hemoglobin (Hb), hematocrit (Ht), mean corpuscular volume (MCV), mean corpuscular hemoglobin (MCH), mean corpuscular hemoglobin concentration (MCHC) and platelet counts were assessed for all subjects. The following serum parameters were analyzed in all subjects: calcium (Ca), inorganic phosphorus (IP), creatinine, albumin, iron (Fe), total iron binding capacity (TIBC), albumin, triglycerides (TG), total cholesterol (T-Cho), high-density lipoprotein cholesterol (HDL), low-density lipoprotein cholesterol (LDL), creatinine (CRTNN), aspartate aminotransferase (AST), alanine aminotransferase (ALT), homocysteine, creatine kinase (CK), lactic acid dehydrogenase (LDH), estradiol (E2), prolactin, thyroid stimulating hormone (TSH), parathyroid hormone (PTH), 25(OH)D, 1,25(OH)_2_D_3_, HbA1c (NGSP), TRACP5b, osteocalcin, uncarboxylated osteocalcin (ucOC), bone alkaline phosphatase (BAP), and P1NP. Urine parameters analyzed in all subjects included uDPD, uNTX, uHydroxyproline and uPentosidine. Calcaneus bone mineral density was analyzed using an ultrasound densitometer (CM-200, Canon Lifecare Solutions Inc., Tokyo, Japan).

### Statistical analysis

Logistic regression analysis was performed in the presence or absence of previous stress fractures, and previous dysmenorrhea, reduced food intake and body weight loss during periods of intense exercise served as independent variables. The strength of an association between stress fractures and the variables was indicated by odds ratios (OR) and their 95% confidence intervals (CI). Data was also analyzed using the Chi-square test. Statistical analysis of serum and urine measurements was undertaken using the unpaired two-tailed Student’s or Welch’s *t*-test (*p < 0.05; **p < 0.01; ***p < 0.001; NS, not significant). All data are shown as means ± S.D. All statistical analyses were performed using Stata 5 software (LightStone Corp, Tokyo, Japan).

## Results

### Dysmenorrhea is associated with stress fractures in female athletes

**Figure 1:**
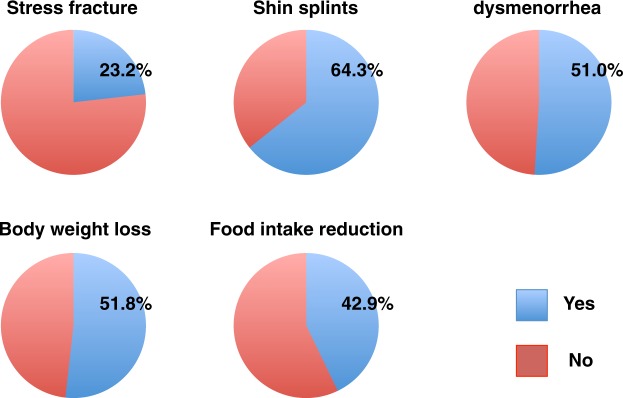
Previous morbidity related to stress fractures in female young athletes. Histories of stress fractures, shin splints, dysmenorrhea, and body weight loss and food intake reduction during high activity periods, as assessed by a self-reported questionnaire.

**Figure 2:**
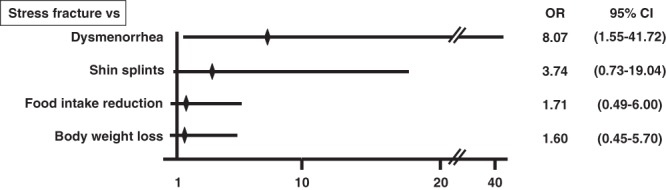
Dysmenorrhea is significantly linked to stress fracture. Odds ratio (OR) and 95% confidence intervals for stress fractures and conditions of dysmenorrhea, shin splints, body weight loss, or reduced food intake, as assessed during the high activity season.

Characteristics of the 56 female athletes are shown in Table 1. Among them, 23.2% had a history of stress fracture (Fig. 1). No subjects were habitual smokers or consumers of alcohol. Among subjects, 64.3% had a history of shin splints, a medial tibial stress syndrome, which is accompanied by tibial pain and a differential diagnosis of stress fracture^25^ (Fig. 1). Also, 51.8, 42.9, and 46.4% had experienced body weight loss, reduced food intake, or dysmenorrhea including amenorrhea, respectively, during prolonged periods of strenuous exercise (Fig. 1). Among conditions reported in histories, dysmenorrhea was significantly associated with stress fractures based on logistic regression analysis (OR, 8.07; 95% CR, 1.55–41.72; p = 0.013) (Fig. 2). Other factors also showed a high OR, but these were not statistically significant (Fig. 2).

**Table 1:** Basic characteristics of subjects.

	Mean	Range
Age (years)	19.9	18–22
Body height (cm)	160.69	149.2–170.9
Body weight (kg)	55.8	39.8–67.8
BMI (kg/m^2^)	21.59	17.3–27.51

### Fracture subjects show increased CK and LDH and decreased osteocalcin

We then subdivided subjects into fracture and non-fracture groups compared various parameters between groups. Basic characteristics such as age, body height, body weight, BMI, age of menarche and bone mineral density (BMD) did not differ statistically between groups (Table 2).

**Table 2:** Basic characteristics of stress fracture and non-fracture subjects.

	Non-fracture	Fracture	*p* value
Age (years)	19.9 ± 0.9	20.2 ± 0.6	0.095
Body height (cm)	160.2 ± 5.5	162.6 ± 4.0	0.139
Body weight (kg)	55.3 ± 5.1	57.3 ± 6.7	0.253
BMI (kg/m^2^)	21.6 ± 1.9	21.7 ± 2.0	0.900
Age of menarche (years)	12.8 ± 1.4	12.4 ± 1.9	0.412
BMD (young adult mean, %)	124.3 ± 19.1	127.5 ± 19.0	0.612

Anemia and iron deficiency are frequently seen in athletes^26,27^. However, complete blood cell counts including red blood cells (RBCs), hemoglobin (Hb), hematocrit (Ht), mean corpuscular volume (MCV), mean corpuscular hemoglobin (MCH), mean corpuscular hemoglobin concentration (MCHC) and white blood cell (WBCs) except platelet counts did not differ between groups (Table 3).

**Table 3:** Measurements in stress fracture and non-fracture subjects.

	Non-fracture	Fracture	*p* value
WBC	6090.5 ± 1742.3	5853.9 ± 1832.9	0.674
RBC	458.8 ± 26.6	448.2 ± 31.2	0.234
Hb	13.7 ± 0.7	13.6 ± 1.1	0.535
Ht	43.5 ± 2.4	43.2 ± 2.4	0.741
MCV	94.8 ± 3.2	96.6 ± 3.4	0.100
MCH	30.0 ± 1.1	30.3 ± 1.5	0.370
MCHC	32.3 ± 4.6	31.4 ± 1.2	0.472
Plt	30.1 ± 4.4	26.8 ± 3.0	0.015
Ca	9.7 ± 0.3	9.8 ± 0.4	0.600
IP	4.1 ± 0.5	3.9 ± 0.4	0.349
Alb	4.8 ± 0.4	4.9 ± 0.2	0.137
T-Cho	197.9 ± 26.4	202.5 ± 30.6	0.599
HDL	77.5 ± 13.1	85.3 ± 11.5	0.060
LDL	108.7 ± 26.5	104.4 ± 25.6	0.608
TG	89.8 ± 56.0	89.5 ± 60.9	0.987
CRTNN	0.70 ± 0.08	0.74 ± 0.11	0.104
Fe	91.1 ± 37.9	96.2 ± 53.7	0.706
TIBC	371.4 ± 47.8	359.5 ± 38.3	0.420
CK	157.2 ± 100.4	266.4 ± 262.8	0.029
AST	21.8 ± 6.3	26.6 ± 13.2	0.073
ALT	16.9 ± 7.0	19.4 ± 7.2	0.270
LDH	184.2 ± 30.2	219.5 ± 52.8	0.004
25(OH)D	23.1 ± 4.9	24.5 ± 4.3	0.360
1,25(OH)_2_D_3_	61.9 ± 13.7	71.1 ± 7.8	0.026

Serum analysis indicated that creatine kinase (CK) and lactic acid dehydrogenase (LDH) levels significantly increased fracture versus non-fracture subjects (Table 3). Although 25(OH)D levels were comparable, levels of 1,25(OH)_2_D_3_, an active form of vitamin D3, were significantly higher in fracture versus non-fracture subjects (Table 3). Levels of other parameters, including calcium (Ca), inorganic phosphorus (IP), Fe, total iron binding capacity (TIBC), albumin, triglycerides (TG), total cholesterol (T-Cho), high-density lipoprotein cholesterol (HDL), low-density lipoprotein cholesterol (LDL), creatinine (CRTNN), aspartate aminotransferase (AST), alanine aminotransferase (ALT), homocysteine and pentosidine, were equivalent between groups (Table 3).

**Figure 3:**
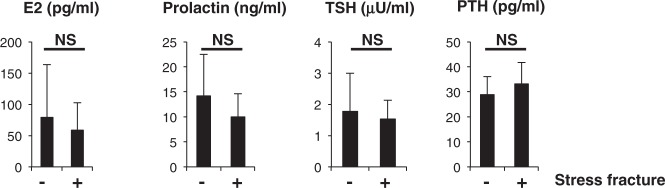
Hormone levels are comparable in stress and non-stress subjects. Indicated hormone levels were determined and compared between stress fracture (+) and non-fracture (−) groups. Data represent mean levels of indicated parameters ± SD (*n* = 43 for non stress fracture, *n* = 13 for stress fracture subjects; NS not significant).

**Figure 4:**
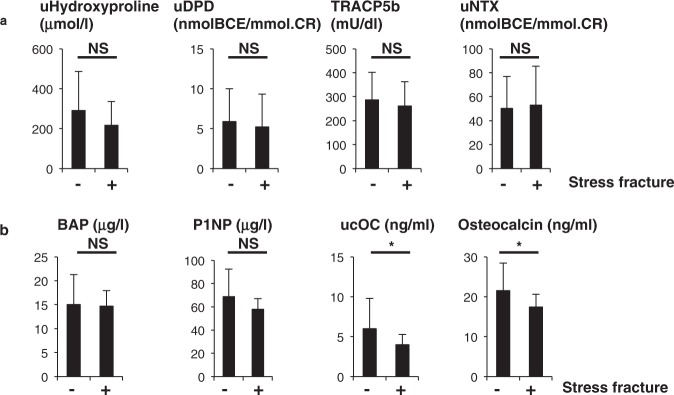
Levels of bone-forming factors ucOC and osteocalcin are significantly lower in stress fracture versus non-fracture subjects. Sera and urine were corrected from stress fracture and non-stress fracture subjects, and levels of indicated parameters were analyzed ((**a**), bone-resorbing parameters; (**b**), bone-forming parameters) and compared between groups. Data represent mean levels of indicated parameters ± SD (*n* = 43 for non stress fracture, *n* = 13 for stress fracture subjects; *p < 0.05, NS not significant). uDPD, urinary deoxypyridinoline; TRACP5b, tartrate resistant acid phosphatase isoform 5b; uNTX, urinary type 1 collagen cross-linked N-telopeptide; BAP, bone alkaline phosphatase; P1NP, procollagen type 1 amino-terminal propeptide; ucOC, undercarboxylated osteocalcin.

Hormonal parameters such as estradiol (E2), prolactin, TSH and PTH levels did not differ between groups (Fig. 3). Assessment of bone metabolic parameters showed that levels of bone-resorbing factors did not differ, but ucOC and osteocalcin, both associated with bone formation, were significantly lower in fracture than non-fracture groups (Fig. 4).

### High exercise activity and stress fracture history are associated with incidence of new stress fractures

Finally, we followed 32 subjects for one year after the first exam. (Other subjects were excluded due to graduation or incomplete data sets.) Among the 32 subjects, three showed new stress fractures during that follow-up period. Follow-up subjects were subdivided into new fracture and non-new fracture groups. Basic characteristics of subjects in both groups are shown in Table 4. There were no statistical differences in age, body height, body weight, BMI or BMD between these groups (Table 4).

**Table 4:** Characteristics of follow-up subjects with or without new stress fractures.

	No fracture	New fracture	*p* value
Age (years)	20.8 ± 0.8	21.3 ± 0.6	0.577
Body height (cm)	161.1 ± 4.7	157.8 ± 2.8	0.234
Body weight (kg)	56.1 ± 5.4	54.5 ± 5.1	0.638
BMI (kg/m^2^)	21.6 ± 1.6	21.9 ± 1.4	0.754
BMD (young adult mean, %)	125.1 ± 20.9	138.3 ± 9.5	0.291

**Figure 5:**
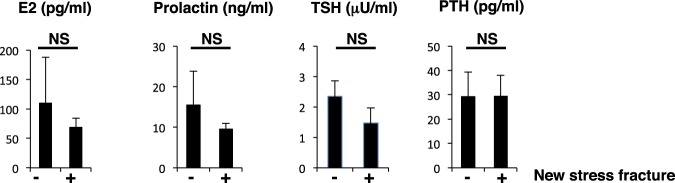
Hormone levels are equivalent in subjects with or without new stress fractures. Levels of indicated hormones were compared between subjects with new stress fracture and those without. Data represent mean levels of indicated parameters ± SD (*n* = 29 for those without new stress fractures, and *n* = 3 for new stress fracture subjects; NS not significant).

**Figure 6:**
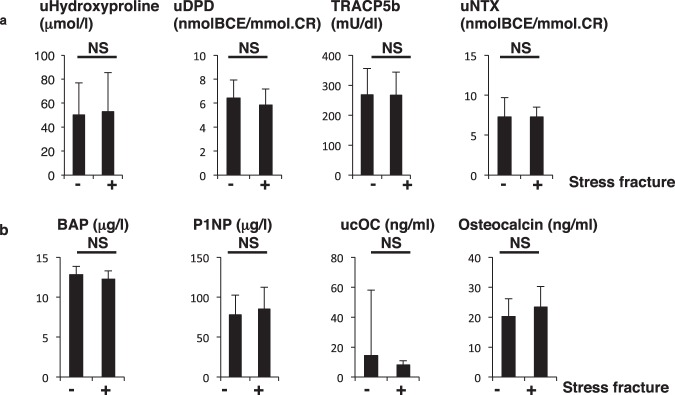
Bone metabolic parameters are comparable in new stress fracture versus non-fracture subjects. Serum and urine samples were corrected from subjects with and without new stress fractures, and levels of indicated parameters were analyzed ((**a**), bone-resorbing parameters; (**b**), bone-forming parameters) and compared between groups. Data represent mean levels of indicated parameters ± SD (*n* = 29 for non-new stress fracture, *n* = 3 for new stress fracture subjects; NS not significant).

Blood tests at follow-up showed that complete blood cell counts, including platelets, and levels of various serum factors did not differ between the two groups (Table 5). Although CK and LDH levels were higher in the group of 3 exhibiting new stress fractures, those levels were not statistically significant, possibly due to the small number of subjects (Table 5). The new stress fracture group showed significantly higher levels of 25(OH)D levels than the non-fracture group (Table 5), but hormonal and bone metabolic parameters were similar between groups (Figs 5 and 6). ucOC levels were lower in the new stress fracture group than the non-fracture group, but those differences were not significant, again possibly due to the limited number of subjects (Fig. 6).

**Table 5:** Measurements in follow-up subjects with or without new stress fractures.

	No fracture	New fracture	*p* value
WBC	5375.9 ± 1409.3	5733.3 ± 2300.7	0.694
RBC	448.1 ± 31.0	460.3 ± 21.0	0.513
Hb	13.3 ± 0.8	13.9 ± 0.3	0.238
Ht	40.6 ± 2.1	42.6 ± 1.2	0.129
MCV	90.8 ± 3.0	92.6 ± 2.3	0.317
MCH	29.7 ± 0.9	30.2 ± 0.8	0.433
MCHC	32.8 ± 0.7	32.6 ± 0.3	0.746
Plt	27.9 ± 5.7	28.1 ± 4.9	0.956
Ca	9.6 ± 0.3	9.9 ± 0.3	0.116
IP	3.9 ± 0.4	3.7 ± 0.2	0.486
Alb	4.9 ± 0.4	5.1 ± 0.4	0.339
T-Cho	196.9 ± 27.4	206.7 ± 10.7	0.549
HDL	81.5 ± 11.2	86.0 ± 11.4	0.515
LDL	101.6 ± 23.0	108.3 ± 10.4	0.621
TG	72.0 ± 43.8	78.3 ± 38.3	0.811
CRTNN	0.75 ± 0.08	0.75 ± 0.05	0.929
Fe	93.0 ± 40.3	90.3 ± 23.6	0.913
TIBC	352.4 ± 40.3	365.0 ± 45.0	0.615
CK	163.1 ± 90.3	209.3 ± 80.3	0.401
AST	23.1 ± 6.5	22.0 ± 2.7	0.775
ALT	16.8 ± 5.6	20.3 ± 6.8	0.312
LDH	178.3 ± 34.9	206.5 ± 24.3	0.192
25(OH)D	20.4 ± 6.0	26.3 ± 1.5	0.030
1,25(OH)_2_D_3_	59.6 ± 23.6	64.4 ± 8.9	0.735

We also asked subjects in the follow-up study about dietary behavior. Caloric intake and nutritional parameters such as intake of protein, fat, carbohydrate, minerals, and vitamins including vitamin D and vitamin K did not differ between groups (Table 6). Moreover, although exercise intensity associated with daily living was similar between groups, exercise intensity associated with sports activity was significantly higher in the new fracture versus the non-fracture group (Table 6).

**Table 6:** Daily nutritional intake or energy consumption values in follow-up subjects with or without new stress fractures.

	Non-fracture	fracture	*p* value
Calories (kcal)	2040.1 ± 652.3	1959.1 ± 448.7	0.856
Water (g)	997.6 ± 316.9	862.0 ± 299.2	0.484
Protein (g)	74.2 ± 19.9	72.1 ± 3.2	0.856
Fat (g)	68.3 ± 16.7	62.1 ± 0.7	0.532
Carbohydrate (g)	269.8 ± 128.9	268.9 ± 105.7	0.991
Sodium (g)	4.1 ± 1.6	2.6 ± 6.9	0.118
Potassium (g)	2.4 ± 0.7	1.9 ± 0.04	0.301
Calcium (mg)	588.2 ± 205.4	462.1 ± 101.3	0.307
Phosphorus (mg)	1086.8 ± 299.1	949.7 ± 35.7	0.441
Iron (mg)	7.9 ± 2.5	7.1 ± 1.1	0.619
Zinc (mg)	9.0 ± 2.7	9.0 ± 1.3	0.987
Vitamin D (μg)	5.0 ± 2.7	3.8 ± 1.2	0.428
Vitamin K (μg)	229.8 ± 104.5	213.9 ± 20.7	0.797
Energy consumption per day by ADL (kcal)	2589.9 ± 712.3	2169.7 ± 284.6	0.325
Energy consumption per week by exercise (kcal)	3019.2 ± 2106.9	7783.0 ± 4934.2	0.008

Finally, 66.6% and 13.7% of new fracture and non-fracture subjects, respectively, had a previous history of stress fracture, a value significantly higher in new stress fracture group based on a chi-square test (X^2^ = 4.99, *p* = 0.026).

## Discussion

Stress fractures occurring in young athletes engaged in vigorous sports activity are due to cumulative low energy stress to bones rather than accidental high energy injuries. Stress fractures not only hamper an athlete’s career but negatively impact their activities of daily living (ADL). Thus, preventing these fractures is mandatory for athletes to continue in their sports and lead normal lives. Bone fragility is thought to precede injury in athletes, and it would be beneficial to predict stress fractures before injury occurs. Low energy availability, functional hypothalamic amenorrhea and osteoporosis are known risks for stress fractures in female athletes. However, no predictive biomarkers are currently available to assess stress fracture risk. Here, we show that elevated CK and LDH, and decreased osteocalcin and ucOC are significantly associated with stress fractures. We also demonstrate that platelet counts were significantly lower in fracture versus non-fracture subjects. We cannot currently explain this result; however, since platelets or associated factors, such as platelet derived growth factor, reportedly function in fracture healing^28^, low platelet counts seen in fracture subjects may account for increased fracture risk. Dysmenorrhea was significantly associated with stress injuries in our cohort, validating our study. In addition, our follow-up revealed that high-intensity sports activity and a history of stress fracture mark athletes at risk. Taken together, we propose that high CK and LDH, low osteocalcin and ucOC, as well as dysmenorrhea, high exercise activity or prior stress fractures are predictive of stress fractures in female athletes.

Our study has some limitations. We analyzed historic but not current stress fracture and dysmenorrhea. Stress fracture is a rare injury, and thus, a large cohort and a means to evaluate stress fracture patients early on are required to analyze status in subjects with current stress fracture. In particular, the number of subjects in our longitudinal follow-up study is very small, making statistical analysis between groups difficult. Moreover, although differences were not significant, CK and LDH were elevated and ucOC levels decreased in the new stress fracture group compared to the group that did not show new stress fractures. Elevated CK and LDH levels likely result from muscle damage, which may occur during strenuous exercise in parallel with fractures. Since these levels were higher in both the fracture versus non-fracture group and in the new fracture versus non-new fracture group, elevated CK and LDH levels could serve as useful markers to predict stress injuries in athletes. To assess this possibility, we performed ROC analysis and found that the cut-off values for each biomarker were CK, 247 U/L; LDH, 199 U/L; osteocalcin, 20.8 ng/ml; and ucOC, 6.29 ng/ml (Table 7). Given the small number of subjects in our study, further analysis is needed to validate results. Nonetheless, monitoring changes in these parameters in athletes during the exercise season could be crucial in predicting stress injuries.

**Table 7:** CK, LDH, OC and ucOC cut-off values as determined by ROC curve analysis.

	CK (U/L)	LDH (U/L)	Osteocalcin (ng/ml)	ucOC (ng/ml)
cut-off value	247	199	20.8	6.29
sensitivity	38.46	69.23	48.78%	36.59%
specificity	90.24	68.29	92.31%	100.0%

Low vitamin D status is reportedly associated with bone fragility fractures in elderly osteoporosis patients^13,14^, and low vitamin D levels are also linked to stress fractures in athletes^15,16^. However, vitamin D levels were higher in fracture than non-fracture subjects in our study. Since vitamin D levels in fracture subjects were <30 ng/ml, these suboptimal levels could also indicate stress injury risk.

Osteocalcin and ucOC levels were significantly lower in non-fracture subjects (Fig. 4). Osteocalcin is produced primarily by osteoblasts and is considered a bone-forming factor, suggesting decreased bone formation in fracture subjects. In general, bone resorption by osteoclasts and bone formation by osteoblasts are regulated in parallel, an activity called coupling. Osteoclasts express estrogen receptors^23^, and estrogen loss at menopause promotes osteoclast activity leading to activated osteoblastic activity in a coupled manner and consequently to reduced bone mass in osteoporosis patients. In our stress fracture subjects, dysmenorrhea including amenorrhea was significantly associated with stress fractures. However, fracture and non-fracture subjects showed similar osteoclastic parameters, while levels of the osteoblast markers osteocalcin and ucOC were significantly lower in the fracture group, suggesting of uncoupling. At present, mechanisms underlying uncoupled regulation of bone status in stress fracture subjects are unclear, but continuous bone and muscle damage by exercise may promote inflammation and oxidative stress, suppressing osteoblastic activity^29,30^.

Osteocalcin is a small protein exhibiting three glutamic acid residues, all carboxylated in a vitamin K-dependent manner to form what is called gla-osteocalcin. Most gla-osteoclacin is trapped in bone matrix, but some is released to the circulation and measured as serum osteocalcin. Vitamin K insufficiency increases serum levels of undercarboxylated osteocalcin, called ucOC, as does osteoclast activity. Fractures stimulate osteoclast activity, elevating serum ucOC in fracture patients. Thus, low ucOC levels can reflect either low osteoblast or osteoclast activity, or low vitamin K intake. However, relative to subjects who did not report new fractures, new stress fracture subjects exhibited comparable osteoclast activity, based on TRACP5b analysis, and vitamin K intake was comparable between groups. The osteocalcin/ucOC system is also hormonally-regulated: ucOC reportedly stimulates insulin secretion from pancreatic beta cells, promoting osteoblast activity^31,32^. Thus, although OC and ucOC were significantly correlated (Table 8), ucOC was considered an independent parameter with osteocalcin, since ucOC played a role in regulating glyco-metabolism, which regulates bone metabolism^32^. However, in our study, HbA1c values, which reflect overall blood sugar levels, did not differ between fracture and non-fracture groups. Furthermore, urinary levels of pentosidine, an advanced glycation end product, were similar between groups, suggesting no changes in glucose metabolism.

**Table 8:** Correlation among CK, LDH, osteocalcin and ucOC.

	CK	LDH	Osteocalcin
LDH	0.681 (*p* < 0.001)		
Osteocalcin	−0.081 (*p* = 0.556)	−0.270 (*p* = 0.047)	
ucOC	−0.100 (*p* = 0.467)	−0.220 (*p* = 0.106)	0.901 (*p* < 0.001)

In our study, 23.2% and 46.4% of subjects experienced previous stress fractures and dysmenorrhea including amenorrhea, respectively, during periods or seasons of high sports activity. These outcomes strongly suggest that educational interventional to prevent stress fractures is necessary for university athletes. Increasing energy intake or decreasing exercise activity is a first line of defense against stress injuries^1,8,9^. Hormonal intervention is a second, but in our subjects, only two of 56 subjects, both of whom had previous dysmenorrhea, took low doses of steroid hormones prior to this analysis, one for one week and the other for two months. Neither were administered steroids of any kind in the course of this study.

Preventing stress fractures first requires that athletes and coaches know who is at risk. We propose that high CK and LDH, and low osteoclacin and ucOC, combined with dysmenorrhea, high exercise activity or prior stress fractures, can predict that risk. Monitoring menstruation status and levels of these serum factors, particularly in high risk athletes with prior stress fracture, at times of high activity could be useful to inform athletes and coaches about an individual’s risk of stress injury.
